# Marketing Archive Management of Drama Intangible Cultural Heritage Based on Particle Swarm Algorithm

**DOI:** 10.1155/2022/6679237

**Published:** 2022-07-07

**Authors:** Cenxi Li, Boya Liu

**Affiliations:** ^1^Sichuan Conservatory of Music, Chengdu 610000, China; ^2^Chongqing Normal University, Chongqing 401331, China

## Abstract

Each Chinese region has its own ancient opera, which is a treasure of folk culture and a living fossil for studying the historical origins of a local culture. It has significant academic value and historical significance at the national and local levels, whether from the perspective of promoting and disseminating national culture or from the perspective of protecting the world's intangible cultural heritage. Based on this practical significance, this paper conducts a study using the particle swarm algorithm to manage the marketing archives of drama intangible cultural heritage. The article employs the PSO algorithm to test the particle swarm optimization algorithm's convergence and conditions. The effectiveness of the algorithm is analysed in the Sphere function, Rosenbrock function, Griewanks function, and Rastrigin noncont function, and then the algorithm is compared, including the calculation speed comparison between the algorithm in this paper and the three optimal fitness functions. The experimental results show that the PSO algorithm has the highest four items in the statistics of the Schwefel function experimental results. About 45.0379 is the best value and 70.5878 is the maximum precision. The optimal average value is 6.1524, while the average value is 56.15245. In comparison to the QPSO and PSO algorithms, the algorithm in this paper has a faster convergence speed and better search accuracy. The topic of the intersection of the disciplines of drama, intangible cultural heritage marketing, and archive management using the particle swarm algorithm is well-developed.

## 1. Introduction

Intangible cultural heritage is the precious cultural wealth of mankind. It is the embodiment and memory of national cultural traditions. It can promote the inheritance of traditional culture and the protection of cultural diversity. Nowadays, more and more people organically combine the protection of intangible cultural heritage with tourism development, attracting the emerging elements of intangible cultural heritage into tourism activities, which has become an important way for intangible cultural heritage to meet the needs and development of modern society. Various forms of folk performances, including folk activities and performing arts, are constantly being updated and gradually integrated into this cultural heritage with great potential for development. As intangible cultural heritage, its scope has a definition, including poetry, mythology, etc. However, it is not limited to this category. In addition, there are many kinds of folk performing arts, such as epics and stories. Even rituals passed down from generation to generation by ordinary people and practices related to traditional folk knowledge and cultural sites are included. For example, the electronic file information management module includes four business modules of file maintenance, review, query, and statistics.

In recent years, China is committed to the protection of intangible cultural heritage, and many experts and scholars have carried out research on this aspect. Hammou I considered that intangible heritage is increasingly featured in the planning of several agencies that aim to raise awareness among countries about safeguarding their cultural diversity and to help them develop programmes so as to protect and preserve this type of heritage. However, there was a lack of information when he solved this problem [1]. Zhang and Lee explored the dialectical relationship between authenticity and alienation in intangible cultural heritage (ICH) tourism research based on assessing the role of cultural values in Chinese tourism. His research has certain implications for tourism marketing, aiming to understand the authenticity of the supply side of intangible cultural heritage tourism. However, his research data is not processed by mixed methods, and there may be data errors [2]. Zhang et al. proposed a tourist-based authenticity model to address this problem, where the authenticity of heritage sporting events contains both “cool” and “hot” factors. Using Naadam as an example, the model examined these factors and their impact on tourist satisfaction and loyalty. About 700 questionnaires were distributed in six locations from eastern to western Inner Mongolia, China. His research showed that there are two separate factors for coldness and heatness. Both factors of cold authenticity directly affect thermal authenticity and satisfaction [3]. Veghe explored the links between cultural heritage, marketing and local community sustainability based on secondary data on engagement, perceived importance, access and stewardship. He argued that despite the increasing recognition and expansion of capitalization, the use of tangible and intangible cultural heritage as assets that may benefit local communities remains limited. However, his research did not involve the different geographical conditions at home and abroad, and has geographical limitations [4]. Although the research content is sufficient, the research on intangible cultural heritage is still limited by geographical and human resources, which makes people in the era of big data and algorithms cannot help considering: Is it possible to manage the intangible cultural heritage archives using the ideas of the intersection of disciplines?

Particle swarm optimization is a hot research topic recently, and people have tried to use this algorithm to study many aspects. Jain et al. reviewed the research progress of particle swarm optimization algorithm (PSO) from 1995 to 2017. He discussed the progress, improvement, and application of particle swarm optimization algorithm. He divided PSOs into nine categories according to different aspects. However, his research did not hybridize PSO technology with other evolutionary technologies, which did not have theoretical breadth [5]. Tang and Guan took time delay as an additional parameter and gave parameter estimates for chaotic systems with time delays. The parameter estimation problem is transformed into an optimization problem, which finds an optimal parameter combination, that is, minimizes the objective function [6]. Chen proposed a multiple attribute decision-making (MADM) method based on interval-valued intuitionistic fuzzy-weighted geometric mean (IIFWGA) operator, interval-valued intuitionistic fuzzy-valued precision function (IVIFV), and particle swarm optimization (PSO) techniques. Among them, the attribute weight and the evaluation value of the alternatives relative to the attribute are denoted by IVIFV. But his research did not generate optimal weights for attributes, which has limitations [7]. Gong Y J first developed a new framework to organically combine PSO with another “learning” optimization technique, leading to the generalized paradigm of “PSO learning”. He also proposed a new ^*∗*^L-PSO algorithm, called genetic learning PSO (GL-PSO), using genetic evolution techniques to generate promising samples for particle swarm optimization (PSO) algorithms. But his study did not perform mechanical parallel stacking [8]. In the research of these scholars, it is inevitable to encounter the limitations of particle swarm optimization. The algorithm is very easy to “premature,” and the population diversity is lacking in the late iteration, which makes it easy to fall into the local optimum situation. This is very unfavorable for the analysis of the problem.

Since its inception, the PSO algorithm has attracted the attention of many researchers due to its few parameters, simple operation, and easy implementation. In a very short period of time, a large number of research findings have emerged. Due to some flaws in the PSO algorithm, relevant scholars have made a number of enhancements, which are primarily focused on the following three aspects: (1) improvements to the PSO algorithm, such as inertia factor and parameter selection; (2) integrating PSO with other optimization algorithms to improve; and (3) optimising the PSO algorithm's network topology. The PSO algorithm has been improved in recent years. Adding the weight w, for example, can achieve the goal of balancing particle local search and overall search ability, which can improve the PSO algorithm's performance. Another example is the dynamic inertia weight change strategy, which uses two attractor and repulsion processes to optimise particle evolution and speed up the algorithm's convergence. It accelerates population convergence and effectively prevents particles from settling in local best-fit solutions. The goal of the article is to improve the algorithm's shortcomings and apply it to the creation of drama intangible cultural heritage archives.

## 2. Particle Swarm Optimization and Intangible Cultural Heritage Deconstruction Methods

### 2.1. Swarm Intelligence Overview

**Figure 1:**
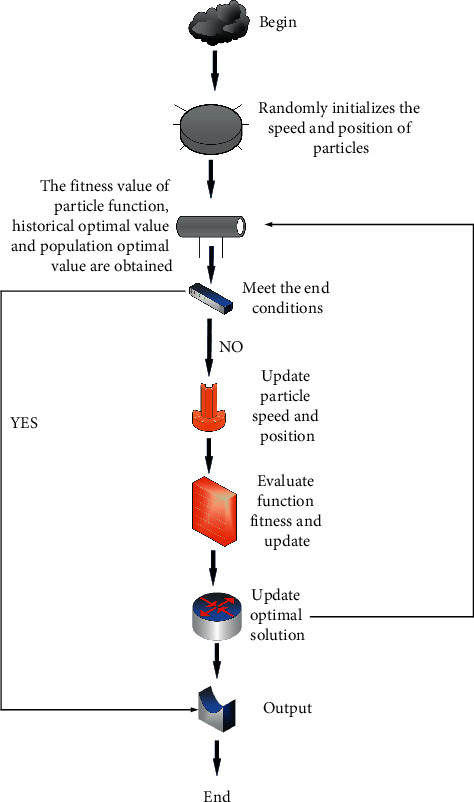
Initial PSO flowchart.

In recent years, team intelligence has received extensive attention in many fields [9, 10]. In nature, although many biological individuals are simple and without intelligence, the behavior of groups is often very complex [11]. This natural phenomenon has aroused great interest of biologists and computer scientists at home and abroad [12]. In recent years, with the rise of biomimicry and research, scientists have begun to study social organisms, such as ants, schools of fish, and flocks of birds, so as to solve practical problems, and swarm intelligence algorithms were born. The swarm intelligence algorithm is a stochastic optimization algorithm that solves practical problems by simulating the intelligent characteristics of certain swarm behaviors in nature [13]. The main characteristics of swarm intelligence include the followings: 1. simplicity; 2. distribution; 3. robustness; 4. wide applicability. Adaptability means that the algorithm proposed in this paper can not only solve the problem of numerical optimization, but also solve the problem of the distribution of discrete variables. It can be a single set of data or a combination of many discrete variables. The initial flowchart is shown in Figure 1:

### 2.2. Explanation of Correlation between the Coordinated Development of Intangible Cultural Heritage and Ecotourism

Since the end of the last century, China's intangible cultural heritage has been dying. The main reasons include two aspects: (1) Many traditional intangible cultural heritage heirs are getting old, and young people are reluctant to learn, resulting in the inheritance of intangible cultural heritage culture. (2) With economic construction and development, many intangible cultural heritage sites have been demolished, resulting in fewer places for cultural heritage. Different from nature tourism, ecotourism is characterized in that it has certain functions of ecological environment education and protection of local characteristic culture [14], which can help tourists and local communities to establish awareness of natural and humanistic environmental protection. It makes tourists have a certain sense of responsibility for the environment and reduce the adverse impact on the environment of ecotourism areas [15], making tourists consciously maintain the stable state of the ecosystem and local culture. The employees of the scenic spot need to take the sustainable development of the scenic spot as the working principle, strengthen the awareness and habits of saving and protection in their work, and infect tourists with practical actions [16], as well as use the cultural heritage of intangible cultural heritage to attract tourists. Intangible culture is closely related and inseparable from traditional Confucianism in the inheritance of thousands of years. It can be said that intangible cultural heritage is a carrier of Confucianism.

Coordination refers to the phenomenon in which two (or more) systems or system factors influence each other through different interactions [17]. This is a benign relationship between multiple systems or elements. “Development” refers to the process in which the system itself or system elements change from small to large, from simple to complex, from low to high, and from disorder to order [18]. “Coordinated development” seems to be based on a virtuous circle, in which the system or system factors develop from low to high, from simple to complex, and from disorder to order. It emphasizes the comprehensive integrated development of multiple systems or systemic factors under the constraints and regulations of “coordination,” seeking global optimization, structural optimization, and joint development based on overall improvement [19]. The degree of coordinated development includes the system or element coordination system (that is, collaborative planning) and the level of development of both [20].

Initiative refers to the principle that the initiative stipulates that collectors of intangible cultural heritage archives must have or cultivate a high sense of responsibility to improve their own enthusiasm and initiative, and have a spirit of loyalty.

### 2.3. Particle Swarm Optimization Algorithm

**Figure 2:**
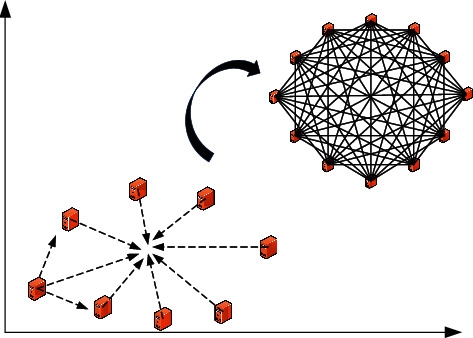
PSO algorithm principle and ER network structure diagram.

**Figure 3:**
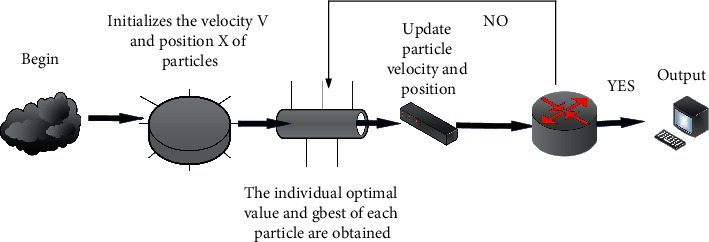
Running process of the standard particle swarm algorithm.

Particle swarm optimization is a random search algorithm. Its evolution equation is a discrete dynamic system equation. By analyzing the state change of the dynamic system, the necessary and sufficient conditions for the algorithm to converge are determined, which provides a theoretical basis for the improvement of the algorithm. The PSO optimization algorithm was originally inspired by the life patterns of flocks of birds and fish. In nature, in the process of foraging, birds sometimes need to forage in groups. Sometimes they also need to forage in groups. In each search, there is always a bird with a strong ability to detect the location of the food, so it will transmit information between the flocks and then guide the flock to the food source. The principle of the PSO algorithm and the structure of the ER network are shown in Figure 2:

Information fusion is an information processing process that automatically analyzes and integrates multisource information through certain rules to achieve prediction, reasoning, and decision-making tasks. Through the above research, the operation process of the standard particle swarm algorithm can be given, as shown in Figure 3.

In order to verify the convergence and conditions of the particle swarm optimization algorithm, the evolution equation of the standard particle swarm optimization algorithm is used for analysis, as shown in the following formulas:

(1) $$\begin{array}{c} V_{\text{id}}\left(t+1\right)=\omega V_{\text{id}}\left(t\right)+c_{1}r_{1}\left({\text{pbest}}_{\text{id}}-x_{\text{id}}\left(t\right)\right)+c_{2}r_{2}\left({\text{gbest}}_{d}-x_{\text{id}}\left(t\right)\right), \end{array}$$

(2) $$\begin{array}{c} x_{\text{id}}\left(t+1\right)=x_{\text{id}}\left(t\right)+v_{\text{id}}\left(t+1\right). \end{array}$$

First, the *d* in the evolution equation is removed, and the particle swarm optimization problem is formulated as a one-dimensional optimization problem, namely as formulas (3) and (4):

(3) $$\begin{array}{c} V_{i}\left(t+1\right)=\omega V_{i}\left(t\right)+c_{1}r_{1}\left({\text{pbest}}_{i}-x_{i}\left(t\right)\right)+c_{2}r_{2}\left(\text{gbest}-x_{i}\left(t\right)\right), \end{array}$$

(4) $$\begin{array}{c} x_{i}\left(t+1\right)=x_{i}\left(t\right)+v_{i}\left(t+1\right). \end{array}$$

In the form of a matrix vector, formula (5) can be obtained as follows:

(5) $$\begin{array}{c} \left[\begin{array}{c} V_{i}\left(t+1\right)\\ x_{i}\left(t+1\right) \end{array}\right]=P\left[\begin{array}{c} V_{i}\left(t\right)\\ x_{i}\left(t\right) \end{array}\right]+Q\left[\begin{array}{c} p_{i}\\ p_{s} \end{array}\right]. \end{array}$$

Among them, there is formula (6) as follows:

(6) $$\begin{array}{c} P=\left[\begin{array}{cc} \omega & -\omega \\ \omega & 1-\phi \end{array}\right],\\ Q=\left[\begin{array}{cc} \phi_{1} & \phi_{2}\\ \phi_{1} & \phi_{2} \end{array}\right]. \end{array}$$

Formula (5) is a discrete-time linear system of equations. Therefore, a sufficient and necessary condition for the algorithm to converge is that the modulus of the eigenvalues of P is less than 1 as follows:

(7) $$\begin{array}{c} f\left(\lambda \right)=\lambda E-P=\left[\begin{array}{cc} \lambda -\omega & -\omega \\ \omega & \lambda -\left(1-\phi \right) \end{array}\right]. \end{array}$$

It can be simplified as follows:

(8) $$\begin{array}{c} \lambda^{2}+\left(\varphi -1-\omega \right)\lambda -\omega =0. \end{array}$$

The eigenvalues of P can be obtained as follows:

(9) $$\begin{array}{c} \lambda =\frac{\varphi -1-\omega \pm {\left(\varphi -1-\omega \right)}^{2}}{2}=0. \end{array}$$

For the convenience of discussion, if the modulus of the eigenvalue is less than 1, formula (10) can be obtained as follows:

(10) $$\begin{array}{c} -2<\omega +1-\phi +{\left(\varphi -1-\omega \right)}^{2}-4\omega <2. \end{array}$$

In order to test the effectiveness of the algorithm, several functions are selected for comparison.

(1) Sphere function
  (11) $$\begin{array}{c} f\left(x\right)=\sum_{i=1}^{n}x_{i}^{2}. \end{array}$$
(2) Rosenbrock function
  (12) $$\begin{array}{c} f\left(x\right)=\sum_{i=1}^{n-1}\left(100{\left(x_{i}^{2}-x_{i+1}^{2}\right)}^{2}+{\left(x_{i}-1\right)}^{2}\right). \end{array}$$
(3) Griewanks function
  (13) $$\begin{array}{c} f\left(x\right)=\sum_{i=1}^{n}\frac{x_{i}^{2}}{4000}-\cos\frac{x_{i}}{i}+1. \end{array}$$
(4) Rastrigin_noncont function
  (14) $$\begin{array}{c} f\left(x\right)=\sum_{i=1}^{n}\left(z_{i}^{2}-10\;\cos\left(2\pi z_{1}\right)+10\right),\\ z_{1}=\left\{\begin{array}{cc} x_{1}\;\;\;x_{1}<1/2,\\ \text{round}\left(2x_{i}\right), & x_{1}\geq 1/2\\ 2, \end{array}\right.. \end{array}$$

In order to change such limitations, generalized transition distributions and acceptance criteria can be used, which can greatly reduce the problem of lack of diversity. In summary, the speed update formula proposed in this paper is as follows:

(15) $$\begin{array}{c} V_{\text{id}}\left(t+1\right)=\omega V_{\text{id}}\left(t\right)+c_{1}r_{1}\left({\text{Pbest}}_{\text{id}}-x_{\text{id}}\left(t\right)\right)+c_{2}r_{2}0.7\times \left(p_{b}-X_{\text{id}}\left(t\right)\right)0.2\times \left(p_{m}-x_{\text{id}}\left(t\right)\right)+0.1\times \left(p_{w}-x_{\text{id}}\left(t\right)\right), \end{array}$$

(16) $$\begin{array}{c} x_{\text{id}}\left(t+1\right)=x_{\text{id}}\left(t\right)+V_{\text{id}}\left(t+1\right). \end{array}$$

## 3. Experiment on the Archives' Management System of Drama Intangible Cultural Heritage

### 3.1. Data of Intangible Cultural Heritage Archives

**Figure 4:**
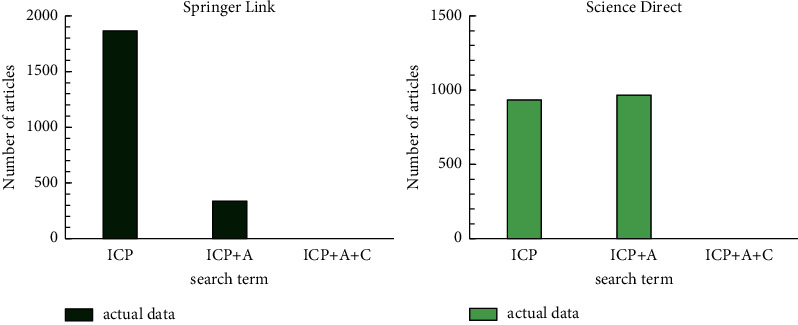
Springer link and science direct literature search results.

At present, the full-text electronic journals provided by Springer link include a total of 439 academic journals. The journals published by Science Direct are recognized as high-quality academic journals in the world, such as the well-known Science journals. Most of them are core journals and are included in many famous secondary literature databases in the world. A data survey was carried out on the research results of foreign cultural heritage archives. ICP stands for “intangible cultural heitage protection.” ICP + *A* means “intangible cultural heitage protection + archives,” and ICP + *A* + C represents “intangible cultural heitage protection + archives + classification.” The result is shown in Figure 4.

As can be seen from Figure 4, there are more documents on intangible cultural heritage archives in Springer Link, but there are no documents on intangible cultural heritage protection + archives + classification in the two libraries.

The CNKI journal database was used to search the full-text database of master and doctoral dissertations in China. The results are shown in Table 1.

### 3.2. Distribution Characteristics of Drama Intangible Cultural Heritage in a Province

**Figure 5:**
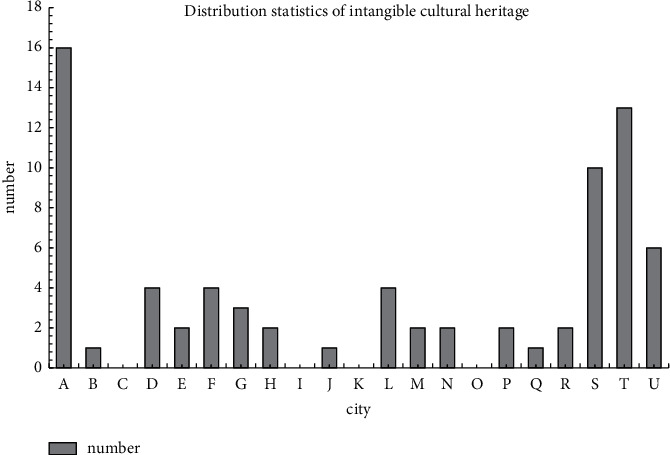
Statistics on the distribution of national intangible cultural heritage in the province.

When studying the distribution of intangible cultural heritage in a province, statistics were made on the regional distribution of national and provincial intangible cultural heritage in the province, which are expressed from A to U according to the ranking of prefecture-level cities in the province, as shown in Figure 5.

It can be seen from Figure 5 that the total amount of intangible cultural heritage in the province is relatively large, but the regional distribution is uneven. There is a positive correlation between the number of national intangible cultural heritage and the regional distribution.

**Figure 6:**
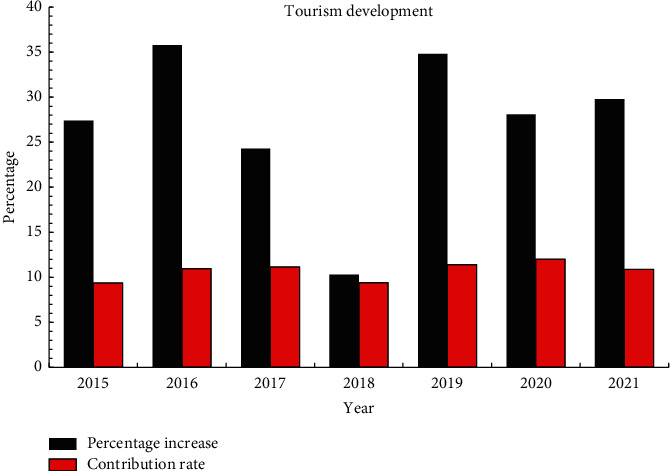
Map of tourism development in the province.

Since the culture of the province is the cultural basis for the survival of traditional drama, the intangible cultural heritage of the province mainly focuses on drama. From 2015 to 2021, the development of tourism to the province due to intangible cultural heritage such as drama is shown in Figure 6.

As can be seen from Figure 6, the province's year-on-year growth rate of intangible cultural heritage tourism revenue has changed from 27.4% to 29.8%, and the proportion of its contribution to the country's tourism has also increased from 9.38% in 2015 to 10.88% in 2021. It shows that the protection of intangible cultural heritage can promote the development of tourism in this area.

**Figure 7:**
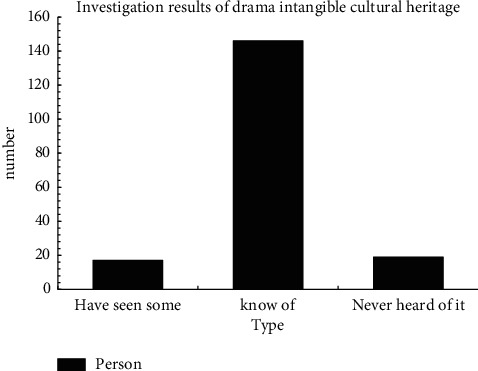
Results of the investigation on the intangible cultural heritage of drama.

A survey was conducted on tourists and ordinary citizens on the intangible cultural heritage of drama. The results are shown in Figure 7.

For plays unique to the province, 17 had seen some, 146 had heard of it but had not seen it, and 19 had not heard of it. It shows that the popularity of the drama is average. About 94% of people have not seen it, while only 6% of people have seen it. Therefore, the drama should be brought into the life of the masses, so that more people have the opportunity to enjoy the drama.

### 3.3. Simulation Test Results of the Intangible Cultural Heritage Archives' Management System

In order to show the performance of different algorithms more intuitively, PSO, DRWS-PSO, and ST-PSO were compared and the three algorithms were run independently for 50 times. The maximum, minimum, average, and average time for each algorithm were derived, as shown in Table 2.

According to Table 2, it can be seen that for the four functions used for testing, the improved ST-PSO algorithm takes less time, which is more efficient than the traditional PSO and DRSW-PSO. In addition, since the ST-PSO algorithm refers to the scale-free network as the domain structure of particles, the experimental results confirm that its algorithm performance is significantly better than that of PSO and DRSW-PSO.

The best solution and worst value of each algorithm and the average and standard deviation of the optimal fitness function are listed below.

Rosenbrock functions are generally nonconvex functions for testing optimal algorithms, which is also called the banana function. The statistics of the experimental results of the Rosenbrock function are shown in Table 3.

In the experimental results of Rosenbrock function, the optimal value of the PSO algorithm is the highest. The worst value is the lowest. The optimal fitness is moderate, which are 44.7568, 237.248, and 26.4578, respectively.

The statistics of the experimental results of the Griewank function are shown in Table 4.

In the statistics of the experimental results of the Griewank function, the average value of the PSO algorithm is the highest, and the optimal fitness is extremely high. The results are 1.3215 and 1.0145, respectively.

The statistics of the experimental results of the Schwefel function are shown in Table 5.

In the statistics of the experimental results of the Schwefel function, the four items of the PSO algorithm are the highest. The optimal value is 45.0379. The maximum accuracy is 70.5878. The average value is 56.15245, and the optimal average value is 6.1524. Therefore, in general, APSO algorithm has faster convergence speed and better search accuracy than QPSO algorithm and PSO algorithm.

**Figure 8:**
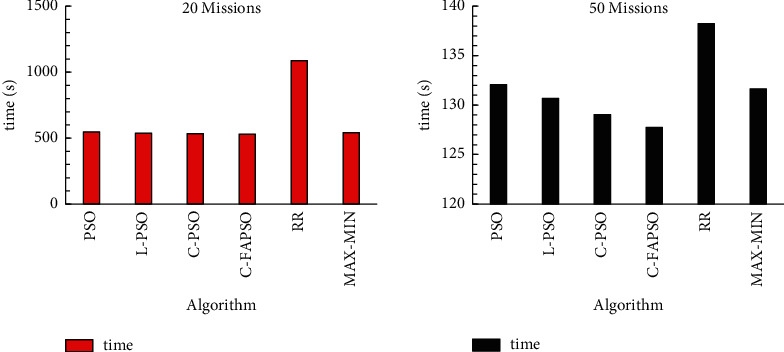
Time spent when there are 20 and 50 tasks.

In order to test the scheduling performance of the improved particle swarm algorithm, the following compares the completion time of the Chebyshev dynamic chaotic perturbation particle swarm optimization algorithm with adaptive inertia weight and traditional particle swarm optimization algorithm, round-robin scheduling algorithm (RR), and Max-Min algorithm. When the tasks are 20 and 50, the comparison results are shown in Figure 8.

Compared with the traditional particle swarm optimization algorithm, the traditional chaotic perturbation particle swarm optimization algorithm, and the chaotic perturbation particle swarm optimization algorithm, the improved particle swarm optimization algorithm converges faster in the repeated initial stage. However, in the later stage of algorithm repetition, the improved algorithm has better convergence effect, which is more stable. It has a shorter total completion time, which obviously meets the needs of users.

**Figure 9:**
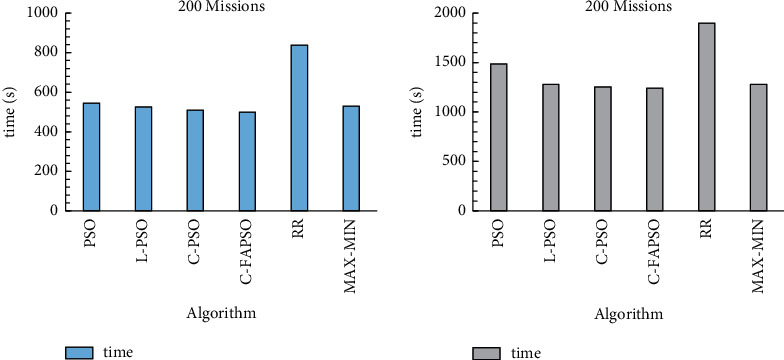
Time spent when there are 200 and 500 tasks.

In order to expand the task volume, the algorithms are compared when the tasks are 200 and 500. The comparison results are shown in Figure 9.

It can be seen from Figure 9 that the Chebyshev chaos perturbation particle swarm optimization (Chevbyshev-FAPSO) with adaptive inertia weight has better scheduling results, and the improved particle swarm optimization takes the shortest time to complete the same tasks.

## 4. Particle Swarm Algorithm and File Management

### 4.1. Agent Modeling and Chaos Mapping

Agent often refers to a person who has the ability to recognize and practice in the objective world. For machines, it is an intelligent body with autonomous perception, judgment, reasoning, and decision-making. From the perspective of knowledge, the Agent is defined as an agent who has experience knowledge and can use knowledge to solve problems. The characteristics of Agent determine that the system it builds is nondeterministic, because the autonomy of Agent will make the system also have autonomy. Such a system is difficult to accurately predict, evaluate, and make decisions. Similarly, the behavior and state of the Agent system are random in design, and it is difficult to determine. Although the behavior of each Agent can be standardized, the system will only show the randomness of the state when it is running. Therefore, the unpredictability of the behavior of the traditional Agent model is a problem that needs to be solved.

Although the Agent method has inherent defects, it is necessary to expand and enhance the interactive semantic representation ability of Agent. The cyber-physical system (CPS) can provide this function to make up for the defect of the Agent system. CPS includes system engineering such as environment perception, embedded computing, network communication, and network control, so that the Agent system has the functions of autonomous perception, communication interaction, judgment, reasoning and decision-making, remote cooperation, and autonomy. Such a CPS-Agent system can accurately predict, evaluate, and make decisions based on the perception of itself, the environment and other agents, which can also solve the real-time dynamic randomness problem of the system.

Chaos covers almost every branch of the natural and social sciences. It has its own unique characteristics. The phenomenon of chaos seems to be random, but it has its inherent delicate structure, instead of intricate and disorganized chaos, which is a variable that fluctuates in the interval [0, 1]. In recent decades, gratifying results have been achieved in the study of chaos theory. Approximation methods and numerical integration methods of nonlinear differential equations have enabled people to fully and thoroughly understand, understand and apply the chaos.

The chaotic variable is ergodic, that is, it can search every state of the space without repetition. The randomness of a chaotic variable means that the turbid variable is as cluttered as a random variable. Chaotic variable is very sensitive to the initial conditions, and a small change in the initial value will cause a huge change in the later movement, which may lead to a large discrepancy. Due to these characteristics, the turbidity-based search technology is more advantageous.

### 4.2. File Management

In recent years, network management and automated document management have quietly become research directions in related fields. Meanwhile, good progress has been made in management level and innovation in theory and practice. For example, developed countries in Europe and the United States, Australia, and other advanced regions have integrated the research methods of network information document management into the national development planning process, and management tools have also changed. That is, they have their own management plans for the company in the country, from loose management to complex unified centralized management. These changes fully demonstrate that network information management has become an inevitable trend in the development of document management. The development of these documents also affects the management of our country.

Because in the original database table, it is often necessary to input file information, and the file information statistics need to generate monthly loan information. Therefore, multiple queries are required to statistic file information. Eventually monthly files are created and stored in statistic view. In countries such as Europe and the United States, it has been proposed to manage documents and documents through a network management framework. At the same time, it will also combine e-government for effective document and archive management. More than 90% of important official documents will no longer be stored on printed materials and computers. In other words, the information management of network file records has become an effective means of government management. Australia also put forward the strategic idea of network file management, aiming to transform traditional management into a more effective network management tool.

### 4.3. RFID Technology

With the rapid development of electronic technology, electronic file management system came into being. It not only improves the efficiency of work, but also backs up the original paper archives to ensure the safety of the archives. However, electronic files are not suitable for storing all data, and large enterprises and companies have a large number of files, so it is impossible to completely convert all files into electronic storage in a short period of time. Therefore, the management method of physical archives is still irreplaceable. It is very urgent to develop a set of archives management system that can meet the current needs.

RFID (Radio Frequency Identification) technology is a new technology that was born at the end of the last century and has developed rapidly in recent years, which is one of the key technologies of the Internet of Things. RFID technology uses radio-frequency antennas to communicate to achieve the purpose of exchanging data and identifying targets, and integrates signal transmission, automatic identification, radar, and other technologies. It has the characteristics of strong storage capacity, high security, and outstanding antipollution ability, and has been maturely used in various fields of society. RFID systems can be classified according to energy supply mode, operating frequency, and working mode.

China's RFID technology started relatively late, with a lack of core technology, especially in RFID UHF is still in its infancy. In addition, China's RFID technology standards are not unified. The products produced by various manufacturers are quite different, and they are not compatible with each other, which greatly limits the development of RFID technology. But in recent years, China's RFID technology has also made great progress. Among them, the automatic identification system of railway vehicle number (ATIS) is the earliest system using RFID technology in China. Using ATIS can accurately collect the running status data of locomotives and vehicles, such as vehicle number, vehicle number, status, location, and departure time, and can track vehicle movements in real time. At present, China has issued a total of 1.4 billion second-generation ID cards, realizing the large-scale application of RFID technology, and it has also been widely used in mobile payment, public transportation, commodity tracking and anticounterfeiting, and medical and health fields.

### 4.4. Backup and Storage of Intangible Cultural Heritage Archives

Sudden and highly destructive events such as fires, earthquakes, and environmental hazards often seriously threaten the security of archives and affect the order of archives work. The daily management of intangible cultural heritage archives includes the formulation of emergency plans. Through drills and training, the staff's emergency rescue ability can be improved in multiple ways. When emergencies occur, emergency protection techniques can be widely used and effective rescue work can be carried out in time, which can not only prevent the slow response, insufficient rescue, and ineffective response caused by insufficient preparation, but also reduce or even avoid the damage of intangible cultural heritage archives. These emergency measures cannot guarantee that the precious intangible cultural heritage will be avoided from the danger of extinction, so the backup and preservation of the intangible cultural heritage archives should also be implemented. The backup storage of intangible cultural heritage archives mainly includes the implementation of off-site backup storage, and heterogeneous backup storage. The off-site backup strategy of archives refers to making real-time available safe copies of intangible cultural heritage archives in another place. Off-site backup storage can minimize the losses caused by unexpected events, but the disadvantage is that the capital demand is too large. Therefore, off-site backup storage is mainly aimed at particularly important intangible cultural heritage files, for example, microforms of precious paper intangible cultural heritage files, files of traditional skills that are on the verge of being lost, etc.

In addition to off-site backup files, intangible cultural heritage files should also implement heterogeneous backup of intangible cultural heritage files. Heterogeneous backup is to realize the backup of different file forms, and use materials of different textures to store the intangible cultural heritage information, for example, the use of optical discs, magnetic disks, or other materials of different textures for backup, in order to achieve the purpose of backup. Compared with the disadvantage of large demand for storage funds for off-site backup, the cost of heterogeneous backup is low and the difficulty of storage is small. After the heterogeneous backup, the intangible cultural heritage archives have multiple copies, which is helpful for the long-term preservation of the intangible cultural heritage archives.

In addition, there is a cloud backup of intangible cultural heritage files. Cloud backup has the advantages of high storage capacity, high utilization rate, high security performance, and low cost. Cloud backup is mainly suitable for intangible cultural heritage electronic files with high frequency of use and large access volume. It can not only ensure the security of archive data, but also realize automatic management and backup for users of intangible cultural heritage archives, which can be fully used in the utilization of intangible cultural heritage archives.

## 5. Conclusions

The treasure of human society is intangible cultural heritage. It carries a nation's history and culture, as well as the nation's cohesion and centrifugal force, which is vital. Intangible cultural heritage has become increasingly marginalised in modern society as our country's economy has developed rapidly. When the feedback algorithm interacts with a human, the process becomes more complicated, and the accuracy of the feedback algorithm is severely limited. The optimization of particle swarm optimization is critical in order to achieve breakthroughs in both retrieval efficiency and accuracy. The topic of using algorithms to manage the archives of the intangible cultural heritage of drama is relatively new at a time when ideas about the intersection of disciplines are gaining traction. It has been tried by a small number of people, and research on it is scarce. The particle swarm algorithm and file management are examined from this practical perspective in this article. Backup storage, intangible cultural heritage file management, RFID, and other contents are among the features introduced. The particle swarm algorithm has been integrated into the marketing archives management of drama intangible cultural heritage, and the research has been completed to a high standard. When the task volume is 20, 50, or 200, 500, the article compares adaptive inertia weight algorithms, and the experimental results are excellent. For future research and development of this academic hotspot, researchers can try to generalise more intangible cultural heritage archives. The protection of intangible cultural heritage can also be done by individuals.

## Figures and Tables

**Table 1:** Results of domestic literature search.

	File management	Archives protection	Intangible cultural heritage + archives protection	Intangible cultural heritage + archives classification
Journal full text	6	21	88	167
Master's thesis	4	2	6	13
Doctoral dissertation	0	0	1	0

**Table 2:** Function test results.

Test function	Algorithm	Max	Min	Average	Average time
*F*1	PSO	18.6564	21.1245	43.0168	0.98
	DRSW-PSO	17.1547	14.6653	20.1518	0.99
	ST-PSO	16.4155	12.1544	13.2201	0.88
	PSO	14.1524	11.1545	10.1576	1.15

*F*2	DRSW-PSO	24.4587	21.2545	22.1547	1.04
	ST-PSO	34.4154	29.2584	32.4557	1.00
	PSO	18.9694	8.7015	8.4852	1.29

*F*3	DRSW-PSO	19.6673	4.8814	4.3343	1.21
	ST-PSO	18.8685	1.0015	1.1205	1.05
	PSO	615.5458	598.5245	606.2218	1.13

*F*4	DRSW-PSO	374.2545	361.5245	368.7541	1.33
	ST-PSO	330.1545	298.5247	311.2435	0.85

**Table 3:** Statistics of experimental results of the Rosenbrock function.

	Best	Worst	Average	Stdev
PSO	44.7568	237.2487	62.4154	26.4578
DRSW-PSO	44.5247	111.4154	48.4558	9.3457
ST-PSO	32.1547	148.1545	56.4789	36.5847

**Table 4:** Statistics of experimental results of Griewank function.

	Best	Worst	Average	Stdev
PSO	0.0916	4.9451	1.3215	1.0145
DRSW-PSO	0.0223	-1.1147	0.2558	0.1685
ST-PSO	0.0578	0.4935	0.1998	0.05457

**Table 5:** Statistics of experimental results of Schwefel function.

	Best	Worst	Average	Stdev
PSO	45.0379	70.5878	56.15245	6.1524
DRSW-PSO	16.2457	39.2458	24.2415	4.2459
ST-PSO	23.2458	39.2456	30.5487	4.1558

## Data Availability

The data used to support the findings of this study are available from the corresponding author upon request.
